# Case Report: Multiple peripheral nerve demyelinating lesions and cerebrovascular injury which resulted in extensive cerebral infarction in a XLP1 patient without EBV infection

**DOI:** 10.3389/fimmu.2025.1580909

**Published:** 2025-05-30

**Authors:** Jiaxun Li, Dongcan Mo, Luyu Lv, Fuling Huang, Binyan Wu, Chunjuan He, Hao Wu, Wenpeng Pang, Yan Li, Liping Guo, Man Luo

**Affiliations:** ^1^ Department of Microbiology, School of Basic Medicine, Guangxi Medical University, Guangxi, Nanning, China; ^2^ Department of Neurology, The First Affiliated Hospital of Guangxi Medical University, Guangxi, Nanning, China; ^3^ Guangxi Key Laboratory of Thalassemia Research, Guangxi Medical University, Guangxi, Nanning, China; ^4^ Department of Radiology, The First Affiliated Hospital of Guangxi Medical University, Guangxi, Nanning, China; ^5^ Department of Cardiology, Guigang People’s Hospital, Guangxi, Guigang, China; ^6^ Department of Pediatrics, The First Affiliated Hospital of Guangxi Medical University, Guangxi, Nanning, China; ^7^ Key Laboratory of Basic Research on Regional Diseases (Guangxi Medical University), Education Department of Guangxi Zhuang Autonomous Region, Guangxi, Naning, China

**Keywords:** X-linked lymphoproliferative syndrome type 1, multiple peripheral nerve demyelinating lesions, cerebral infarction, SH2D1A gene, case report

## Abstract

X-linked lymphocytic proliferative disease type 1 (XLP1) is a primary immune deficiency caused by genetic alterations in the *SH2D1A* gene, exhibiting a wide variety of severe clinical phenotypes and high mortality. We present the case of a 16-year-old male patient diagnosed with XLP1 who suffered from multiple peripheral nerve demyelinating lesions and extensive cerebral infarction, resulting in two consecutive admissions. The onset of symptoms in his first admission were presented with right foot weakness and difficulty walking. The electromyogram findings revealed multiple peripheral nerve damage of bilateral lower limbs, mainly demyelination. Combined with the cerebrospinal fluid tests and medical history, the patient was diagnosed with chronic inflammatory demyelinating multiple radiculopathy (CIDP). After neurological rehabilitation physiotherapy, vitamin B12 supplements and hormonotherapy, the patient’s symptoms improved and he was discharged. Ten days after discharge, he was readmitted with dizziness, lethargy, memory and cognitive decline. Imaging findings included MRI, Arterial spin labeling (ASL) and magnetic resonance spectral (MRS) confirmed that the patient had suffered from a cerebral infarction, the results of follow-up magnetic resonance angiography (MRA) and magnetic resonance vessel wall imaging were consistent with the typical imaging findings of cerebral vasculitis. No EBV infection was detected during his two admissions, which was extremely rare in XLP1 cases. Due to the patient’s guardians declining the hematopoietic stem cell transplantation (HSCT), we were limited to symptomatic and supportive treatments, focusing on improving brain metabolism, and with a transitory stable condition at present. This case expands our understanding of rare XLP1 complications, shows a potential to study on the immune-related mechanism of possibly lymphocytic abnormal proliferation disorder associated with XLP1 involving both peripheral nerves and cerebrovascular, underscores that XLP1 patients should have regular examinations on their central and peripheral nervous system in order to detect early lesions and prevent serious consequences. And we are able to gain valuable experience and lessons from the patient’s disease progression and prognosis.

## Introduction

X-linked lymphoproliferative disease type 1 (XLP1), an X-linked recessive genetic disorder, is associated with primary immunodeficiency due to genetic alterations of the *SH2D1A* gene encoding the signaling lymphocyte activation molecule (SLAM)-associated protein (SAP) (1). The disease is associated with an increased susceptibility to Epstein-Barr virus (EBV) infection and characterized by hemophagocytic lymphhistiocytosis (HLH), hypogammaglobulinemia, and lymphoma (2). However, with more and more in-depth study of XLP1, the disease has exhibited an increasing number of atypical clinical phenotypes.

The available case reports of XLP tell us that neurologic involvement in the progression of XLP, especially the central nervous system (CNS). A variety of CNS manifestations that may occur in XLP patients have been reported (e.g., CNS vasculitis, diffuse CNS vasculopathy, CNS lymphomas, etc.). Among them, CNS vasculitis related to the patient in this case is a rare clinical manifestation of XLP, which was first reported in XLP in 1985 (3). The pathogenesis of vasculitis in XLP is poorly understood, which may due to the aberrant activation of CD8^+^ lymphocytes by EBV^+^ B cells. Alternatively, vessel walls might themselves become directly infected by EBV due to altered viral clearance (4). The mechanism of non-EBV associated CNS vasculitis in XLP patients remains unknown. Most XLP patients died from their CNS vasculitis. Given its poor outcome, CNS vasculitis in XLP should be recognized promptly and treated aggressively (5). The peripheral nervous system involvement in XLP is still being explored.

In this case, we report an unusual patient who suffered from both multiple peripheral nerve demyelinating lesions and CNS vasculitis possibly caused by lymphocytic abnormal proliferation disorder associated with XLP1, but without evidence of EBV infection or other infections, which was a rare occurrence. After two closely separated admissions, the patient’s condition was basically stable, but the electromyogram findings indicated that his peripheral nerves damage was still severe, with axonal damage appeared. We’ll continue to record his condition to provide a valuable reference of other XLP1 patients’ assessment on their disease progression, prognosis and outcomes whose clinical findings are similar to this case and without undergoing HSCT.

## Case presentation

A 16-year-old boy was admitted to our hospital due to weakness, numbness, and difficulty walking in his right foot for over 3 months, with symptoms worsening in almost half a month. Electromyogram conducted 2 days before admission found abnormal motor and sensory nerve conduction in both lower limbs, and conduction block in proximal stimulation, which suggested multiple peripheral nerve damage in both lower limbs, mainly demyelination (**Table 1a**).

Table 1AElectromyogram results obtained two days before the patient’s first admission.

**Table:** 

Motor NCS							
Nerve/position	Muscle	Latency (ms)	Amplitude (mv)	Segment	Dist (mm)	Lat Diff (ms)	Velocity (m/s)
Right common peroneal nerve							
Ankle	EDB	4.6	0.7	B. Fib Head-Ankle	280	8.2	34
Up-fibulae capitulum	EDB	12.9	0.3	A. Fib Head - B. Fib Head	140	3.0	47
Under- fibulae capitulum	EDB	15.8	0.3	Acc Peron - A. Fib Head			
Right tibial nerve							
Ankle	AH	3.3	3.5	Knee - Ankle	400	9.6	42
Knee	AH	12.9	1.4				
Left common peroneal nerve							
Ankle	EDB	3.2	5.2	B. Fib Head-Ankle	330	7.8	42
B. Fib Head	EDB	11.0	3.4	A. Fib Head - B. Fib Head			
Left tibial nerve							
Ankle	AH	3.3	5.8	Knee - Ankle	380	9.5	40
Knee	AH	12.8	1.9				
Left median nerve							
Wrist	APB	2.6	11.1	Elbow - Wrist	210	4.8	44
Elbow	APB	7.4	7.6	Axilla - Elbow		5.1	
Erbs	APB	12.5	6.3	Erbt’s Pt - Axilla			
Left ulnar nerve							
Wrist	ADM	2.5	10.7	B. Elbow - Wrist	170	3.2	53
B. Elbow	ADM	5.7	9.4	A. Elbow - B. Elbow	100	2.1	48
A. Elbow	ADM	7.8	9.2	Axilla - A. Elbow		5.6	
Erbs	ADM	13.4	6.1	Erbt’s Pt - Axilla			
Left musculocutaneous nerve							
1	Biceps	2.4	8.4				
Left axillary nerve							
Erbt’s Pt	Deltoid	2.5	9.9				
Right median nerve							
Wrist	APB	3.1	13.3	Elbow - Wrist	210	3.6	59
Elbow	APB	6.7	12.9	Axilla - Elbow		5.3	
Erbs	APB	11.9	13.9	Erbt’s Pt - Axilla			
Right ulnar nerve							
Wrist	ADM	2.4	9.4	B. Elbow - Wrist	270	5.3	51
B. Elbow	ADM	7.6	9.0	A. Elbow - B. Elbow		5.1	
Erbs	ADM	12.7	8.9	Axilla - A. Elbow			
Right musculocutaneous nerve							
1	Biceps	2.7	12.4				
Right axillary nerve							
Erbt’s Pt	Deltoid	2.9	13.1

**Table:** 

F Wave			
Nerve	M Latency (ms)	F Latency (ms)	% F
Right common peroneal nerve	5.1	56.5	62.5
Right tibial nerve	4.0	50.8	100
Left common peroneal nerve	3.8	47.2	100
Left tibial nerve	4.2	52.2	100
Left median nerve	2.8	25.1	100
Left ulnar nerve	3.1	25.3	100
Right median nerve	2.9	25.0	100
Right ulnar nerve	2.1	25.9	100

**Table:** 

Sensory NCS						
Nerve	Rec. Site	Lat (ms)	Amp (uV)	Segment	Distance (mm)	Velocity (m/s)
Right superficial peroneal nerve						
Lat leg	Ankle			Lat leg - Ankle		
Right sural nerve						
Calf	Ankle	2.3	1.9	Calf - Ankle	90	39
Left superficial peroneal nerve						
Lat leg	Ankle	2.6	10.5	Lat leg - Ankle	120	46
Left sural nerve						
Calf	Ankle	2.2	2.5	Calf - Ankle	95	43
Left median nerve						
Wrist	Index	2.4	21.5	Wrist - Index	140	57
Left ulnar nerve						
Wrist	Dig V	2.1	25.1	Wrist - Dig V	120	56
Right median nerve						
Wrist	Index	2.6	24.8	Wrist - Index	135	52
Right ulnar nerve						
Wrist	Dig V	2.3	31.1	Wrist - Dig V	125	53

Description:

SCV, The sensory conduction potentials of right superficial peroneal nerve could not be elicited.

The sensory conduction velocity and amplitude of the left superficial peroneal nerve were normal.

The bilateral sural nerve sensory conduction velocity slowed down and amplitude decreased.

The sensory conduction velocity and amplitude of bilateral median nerve and bilateral ulnar nerve were normal.

MCV, The Right common peroneal nerve motor latency was normal, (B. Fib Head-Ankle) nerve conduction velocity slowed down, (A. Fib Head - B. Fib Head) nerve conduction velocity was normal, amplitude was reduced, and conduction block was seen.

The motor latency, conduction velocity, and amplitude of the left common peroneal nerve were normal.

The motor latency and conduction velocity of the right tibial nerve were normal, the amplitude was reduced, and conduction block was seen.

The motor latency and conduction velocity of the left tibial nerve were normal, and the stimulation amplitude at the ankle was normal, the stimulation amplitude at the popliteal fossa was reduced, and conduction block was seen.

The motor latency, conduction velocity and amplitude of bilateral median nerve and bilateral ulnar nerve were normal.

F wave: The F wave latency of right common peroneal nerve was prolonged and its occurrence rate decreased.

The F wave latency and occurrence rate of left common peroneal nerve, bilateral tibial nerve, bilateral median nerve, and bilateral ulnar nerve were normal.

SSR: The latency and amplitude of nerve sympathetic skin response (SSR) of bilateral tibial nerve were normal.

Conclusion: Abnormal conduction of motor and sensory nerves in bilateral lower limbs, and conduction block was seen in proximal stimulation, suggesting multiple peripheral nerve damage in bilateral lower limbs, mainly demyelination. The motor and sensory nerve conduction of bilateral upper limbs were not abnormal.

Electromyogram results obtained from the fifth day of the patient’s second admission.

Table 1BElectromyogram results obtained from the fifth day of the patient’s second admission.

**Table:** 

Motor NCS							
Nerve/position	Muscle	Latency (ms)	Amplitude (mv)	Segment	Dist (mm)	Lat Diff (ms)	Velocity (m/s)
Right common peroneal nerve							
Ankle	EDB	6.8	0.1	B. Fib Head - Ankle	350	8.5	4.1
B. Fib Head	EDB	15.3	0.1	A. Fib Head - B. Fib Head			
Right tibial nerve							
Ankle	AH	4.4	1.6	Knee - Ankle	400	10.9	37
Knee	AH	15.3	0.9				
Left common peroneal nerve							
Ankle	EDB	3.7	2.6	B. Fib Head - Ankle	330	9.6	35
B. Fib Head	EDB	13.3	2.0	A. Fib Head - B. Fib Head			
Left tibial nerve							
Ankle	AH	4.7	3.0	Knee - Ankle	400	10.6	38
Knee	AH	15.3	2.1

**Table:** 

F Wave			
Nerve	M Latency (ms)	F Latency (ms)	% F
Right common peroneal nerve	7.4	47.9	75
Right tibial nerve	5.7	58.8	93.8
Left common peroneal nerve	4.5	50.1	100
Left tibial nerve	4.6	66.2	85.7

**Table:** 

Sensory NCS						
Nerve	Rec. Site	Lat (ms)	Amp (uV)	Segment	Distance (mm)	Velocity (m/s)
Right superficial peroneal nerve						
Lat leg	Ankle			Lat leg - Ankle		
Right sural nerve						
Calf	Ankle	3.4	3.1	Calf - Ankle	110	32
Left superficial peroneal nerve						
Lat leg	Ankle	2.3	3.7	Lat leg - Ankle	90	39
Right sural nerve						
Calf	Ankle	2.6	4.2	Calf - Ankle	95	36

**Table:** 

H Reflex				
Nerve	H Latency (ms)	H Amp pp (mv)	M Amp pp (mv)	H/M amplitude (%)
Right tibial nerve			4.7	
Left tibial nerve			9.9	

Description:

SCV, The sensory conduction potentials of right superficial peroneal nerve could not be elicited.

The sensory conduction velocity and amplitude of left superficial peroneal nerve and bilateral sural nerve decreased.

MCV, The motor latency of the right common peroneal nerve was prolonged, the conduction velocity was slowed, and the amplitude was reduced.

The motor latency of left common peroneal nerve and bilateral tibial nerve were normal, the conduction velocity and amplitude were decreased.

F wave, The F wave latency and occurrence rate of bilateral common peroneal nerve were normal.

The F wave latency of bilateral tibial nerve was prolonged and the F wave occurrence rate of bilateral tibial nerve was normal.

H Reflex, The H reflex of bilateral tibial nerve was not elicited.

SSR, The latency and amplitude of nerve sympathetic skin response (SSR) of bilateral tibial nerve were normal.

Conclusion: Abnormal conduction of motor and sensory nerves in bilateral lower limbs, suggesting multiple peripheral nerve damage in bilateral lower.

limbs, Demyelination and axonal damage.

The patient was born at term to healthy, non-consanguineous parents following an uneventful pregnancy. His birth history, growth history, and vaccination history revealed no significant deviations when compared to those of children of the same age. He had a history of intermittent cough in childhood, which improved after taking cough medication. The patient has a younger brother, healthy and family history revealed no history of immune deficiency, abnormal miscarriages, prenatal abnormalities and stillbirths on the maternal side.

Upon physical examination, the patient was alert and oriented to person, place, and time, with intact self-awareness. Speech was fluent and coherent. Cognitive assessment revealed deficits in both recent and remote memory. Motor examination demonstrated grade 4 muscle strength (Medical Research Council scale) bilaterally in the lower limbs, with generalized hypertonia. Sensory testing showed diminished light touch and hyperalgesia in the right lower limb, while proprioceptive and cortical sensations were intact. Deep tendon reflexes were hyperactive (+++) at both knees (patellar reflexes), with all other reflexes being normal. Plantar responses were flexor bilaterally (negative Babinski sign), and meningeal signs (Kernig’s and Brudzinski’s signs) were negative. Cardiovascular, respiratory, and abdominal examinations showed no abnormalities.

The cranial CT scan conducted on the third day of admission revealed no cerebral hemorrhage and space-occupying lesions. CT angiography (CTA) showed no obvious abnormality of cerebral vessels. The Chest CT scan conducted on the fifth day of admission revealed the middle lobe bronchus of the right lung were slightly narrow. The cranial MR enhancement performed on the seventh day of admission showed normal findings. Electrocardiogram, cardiac, cervical and abdominal ultrasound showed normal findings (not shown).

Laboratory tests were detailed in **Supplementary Table S1**, showing a significant decrease in IgA, which suspected hypogammaglobulinemia. There was a transient increase in C-reactive protein and erythrocyte sedimentation rate (ESR), and the two indicators subsequently decreased to near normal ranges. Other inflammatory markers such as white blood cells, neutrophils, procalcitonin (PCT), ferritin and Interleukin-6 (IL-6) remained relatively low throughout the disease course. The lymphocyte subsets analysis showed that a significant lower percentage of B lymphocytes, NK cells and the absolute count of NK cells than the normal range. Cerebrospinal fluid (CSF) had elevated white blood cell and high protein levels, indicating the possibility of intracranial infection, cerebral vasculitis, cerebral hemorrhage or intracranial tumors. Antibodies tests of CSF-XPert and CSF-GFAP were negative. The patient tested negative for serum antigens or antibodies to streptococcus, cryptococcus, Influenza virus, HBV, HCV, HIV, treponema pallidum, cytomegalovirus and Herpes simplex virus 1/2. Two tests for EB virus DNA and antibodies were negative. Metagenomics next Generation Sequencing (mNGS) in cerebrospinal fluid and bronchoalveolar lavage fluid was negative. Results of serum immunofixation electrophoresis were negative. Antibodies to anti-nuclear, anti-nuclear extract, anti-neutrophil cytoplasmic and anti-cardiolipin were all negative. Meanwhile, a whole exome sequencing (WES) sample was retained.

During the first 18 days after admission, we mainly took neurological rehabilitation physiotherapy and vitamin B12 supplements in order to restore the function of peripheral nerves. However, the CSF-protein was significantly elevated with no infections on the 19th day of admission. Combined with the electromyogram findings and medical history, the patient was consistent with the diagnostic criteria of chronic inflammatory demyelinating multiple radiculopathy (CIDP), and the abnormal results of CSF belonged to a phenomenon of CSF-protein and cells separation. We decided to use methylprednisolone for hormonotherapy (500g/d for 3 days, and 250g/d for 2 days), and there was no adverse reaction in the process. After the above treatments, the patient’s symptoms of weakness in the right lower limb improved, and he was discharged on the 24th day.

**Figure 1:**
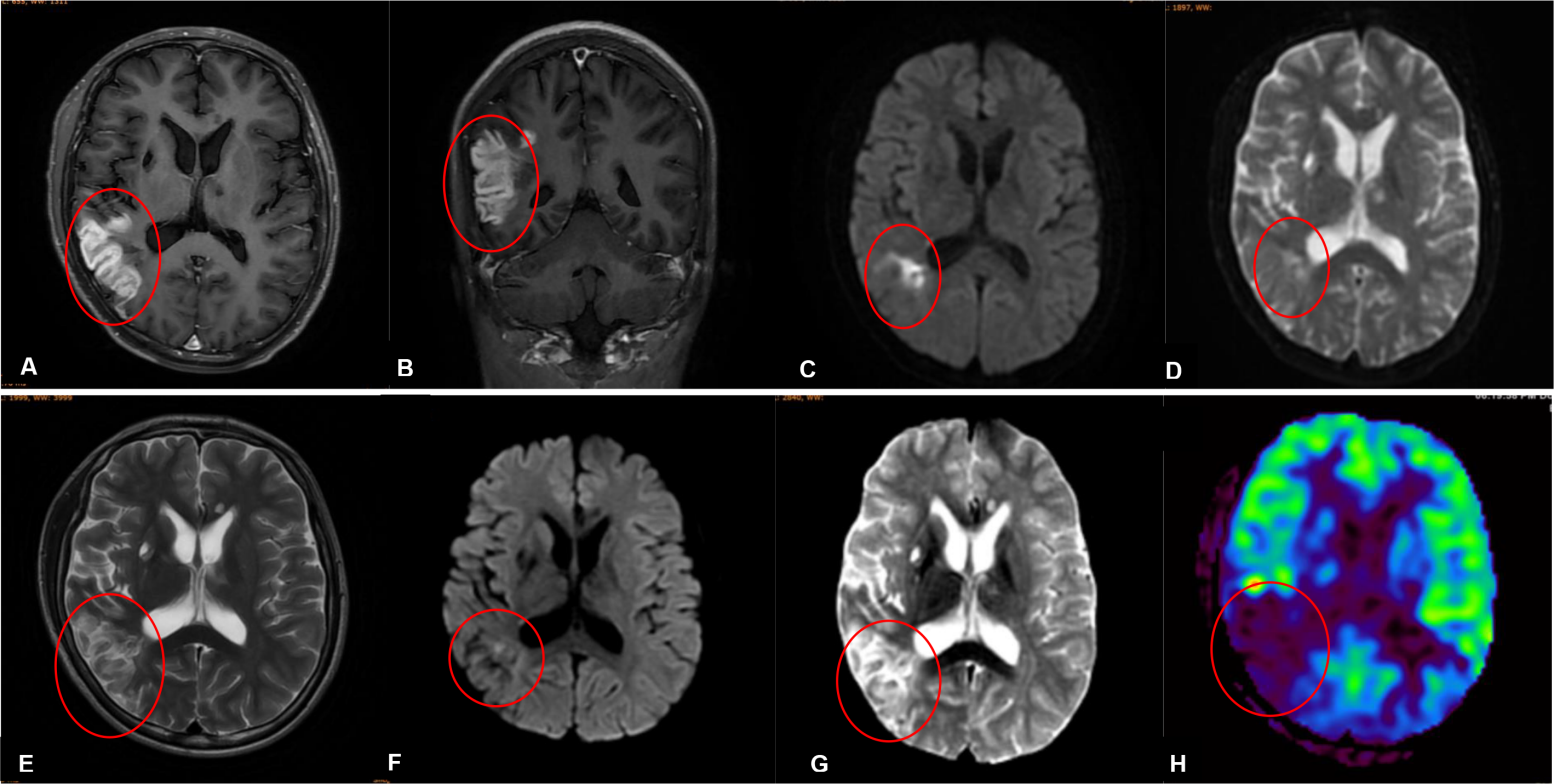
**(A-D)** Imaging findings obtained prior to his second admission. **(E-H)** Imaging findings obtained on the third day of his second admission. **(A, B)** Contrast-enhanced magnetic resonance imaging scans revealed patchy abnormal signals in the right temporal and parietal lobes with significant gyri-like enhancement. **(C)** The DWI in the right temporal and parietal lobes presented high intensities with significant limited diffusion. **(D)** MRI findings on ADC image in the right temporal and parietal lobes presented low intensities. **(E)** MRI findings on the third day of his second admission revealed lessons with high intensities on T2-weighted images in the right temporal and parietal lobes. **(F)** MRI findings on DWI image in the right temporal and parietal lobes presented slightly high intensities with limited diffusion. **(G)** MRI findings on ADC image in the right temporal and parietal lobes presented high intensities. **(H)** Arterial spin labeling (ASL) showed that cerebral blood perfusion in the right temporal, occipital and parietal lobes decreased significant.

Unfortunately, ten days after the discharge, the patient developed dizziness, slurred speech, slow reaction times, reduced cognitive ability, and he was readmitted on the next day. At admission, his body temperature, blood pressure, heart rate and other vital signs were normal, the physical examination results were basically consistent with the first admission. The cranial MR enhancement showed multiple patchy abnormal signals in the right temporal and parietal lobes with significant gyri-like enhancement (**Figures 1A, B**). MRI findings on the DWI image presented high intensities with significant limited diffusion (**Figure 1C**). The ADC image presented low intensities (**Figure 1D**), which indicated an acute phase lesion, and the possible diagnoses considered were cerebral infarction, acute necrotizing encephalitis, cerebral vasculitis. Inflammatory markers were within the normal range, a test for EB virus DNA and antibodies were negative (**Supplementary Table S1**). Due to the cognitive ability decline, the patient did not cooperate with the lumbar puncture examination.

On the second day of admission, a hemizygous variant in the *SH2D1A* gene was detected in the family’s whole exome sequence (**Supplementary Figure S1A**, NM: 002351.5: exon2: c.163C>T, p.R55*, inherited from the mother), which was pathogenic and associated with X-linked lymphoproliferative syndrome type 1 (OMIM:308240). We tried to find the source of the mother’s mutation by Sanger sequencing, and to our surprise, none of the 10 participants found a mutation in this gene locus (the patient’s grandfather had died of natural causes), suggesting that his mother’s mutation may have been spontaneous (**Supplementary Figure S1B**). The patient’s maternal family pedigree was shown in **Supplementary Figure S1C**, his mother and other relatives were not affected by the condition. The mutation type of this patient was stop-gained, which resulted in incomplete production of SAP, and this mutation had a great impact on the structure of SAP (**Supplementary Figures S1D, E**), and might be a possible reason for the development of the observed clinical manifestations in this patient. The mutation information of the SH2D1A gene of the patient and his parents is presented in **Supplementary Table S2**.

MRI findings obtained on the seventh day of admission revealed lessons with high intensities on T2-weighted image in the right temporal and parietal lobes (**Figure 1E**). The DWI image presented slightly high intensities with limited diffusion (**Figure 1F**). The ADC image presented high intensities (**Figure 1G**), which indicated that the lesions had entered the subacute phase. Compared with the MRI findings ten days ago, edema in the right temporal and parietal lobes was more obvious, limited diffusion range of DWI was decreased, with the signal dropped. ASL revealed that cerebral blood perfusion (CBF) in the Partial branches of the middle cerebral artery, right temporal, occipital and parietal lobes decreased significantly (**Figure 1H** and **Table 2**). Magnetic resonance spectral analysis (MRS) showed that the lactic acid peak was inverted in the right temporal and occipital lobes, the NAA peak was lower than that of the opposite side (not shown). These findings supported the diagnosis of cerebral infarction. Electromyogram results obtained on the seventh day of admission demonstrated that the peripheral nerves damage of bilateral lower limbs was aggravated compared with the previous results, with axonal damage appeared (**Table 1b**).

**Table 2:** Cerebral blood perfusion data obtained on the third day of the patient’s second admission.

Cerebrovascular area	Right side (CBF)		Left side (CBF)	
	PLD 1.5s	PLD 2.5s	PLD 1.5s	PLD 2.5s
Anterior cerebral artery A1 area	50.60	45.69	47.16	41.69
Anterior cerebral artery A2 area	38.99	40.91	44.60	43.43
Middle cerebral artery M1 area	56.13	57.01	52.02	48.13
Middle cerebral artery M2 area	31.73	37.13	57.47	49.91
Middle cerebral artery M3 area	15.10	26.56	43.45	48.31
Middle cerebral artery M4 area	43.96	52.28	48.84	44.20
Middle cerebral artery M5 area	39.09	50.46	48.27	45.69
Middle cerebral artery M6 area	17.11	31.54	33.96	41.90
Posterior cerebral artery P1 area	24.28	30.77	26.35	36.15
Posterior cerebral artery P2 area	23.37	39.71	29.08	46.14
Temporal lobe	21.34	32.18	33.90	39.93
Occipital lobe	13.81	21.71	38.36	44.91
Parietal lobe	24.49	42.12	34.82	43.62
Basal ganglia region	44.44	40.80	43.32	36.67
Cerebellar hemisphere	33.91	45.46	31.86	37.09
Pons	42.52	33.49	41.67	23.17

Based on the current findings, without evidence of other specific disease that resulted in the patient’s clinical findings, also in the absence of EBV infection or other infections, we believed that both the patient’s peripheral nerve demyelinating lesions and cerebrovascular injury could be attributed to the immune-mediated accidents which possibly caused by a lymphocytic abnormal proliferation disorder associated with XLP1. We had planned to perform the brain and peripheral nerve biopsies to further confirm this conclusion, but we postponed the plan because of the potential risk of biopsy, especially a peripheral nerve biopsy, which could lead to neurologic deficit symptoms in the patient.

We considered that HSCT might be the most suitable radical treatment, however, his parents refused. Therefore, we could only improve the patient’s brain metabolism with oxiracetam and idebenone until his memory and cognition improved and he was discharged on the 7th day.

**Figure 2:**
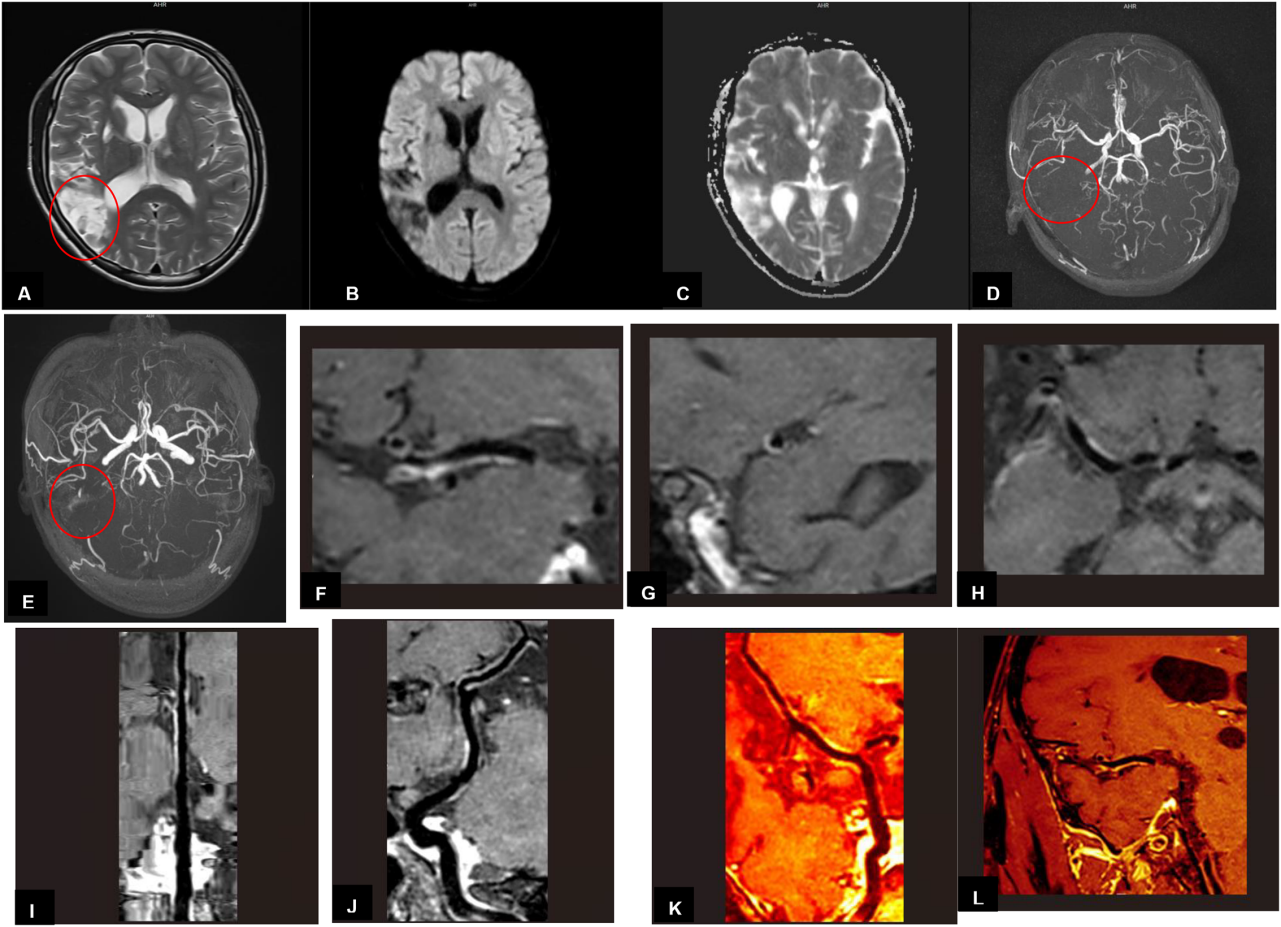
**(A-D)** Imaging findings obtained 8 weeks after the second discharge. **(E-L)** Imaging findings obtained 16 weeks after the second discharge **(A)** MRI findings revealed multiple encephalomalacia foci with high intensities on T2-weighted image in the right temporal and parietal lobes. **(B)** MRI findings on DWI image in the right temporal and parietal lobes presented no limited diffusion. **(C)** MRI findings on ADC image in the right temporal and parietal lobes presented high intensities. **(D)** MRA revealed that the right middle cerebral artery was thinner than that of the left, with fewer branches. **(E)** MRA showed a thickened vascular shadow beside the M1 bifurcation of the right middle cerebral artery, its distal end and branches were not visualized. **(F-L)** Magnetic resonance vessel wall imaging showed homogeneous concentric thickening with enhancement in the M1 segment of the right middle cerebral artery.

**Figure 3:**
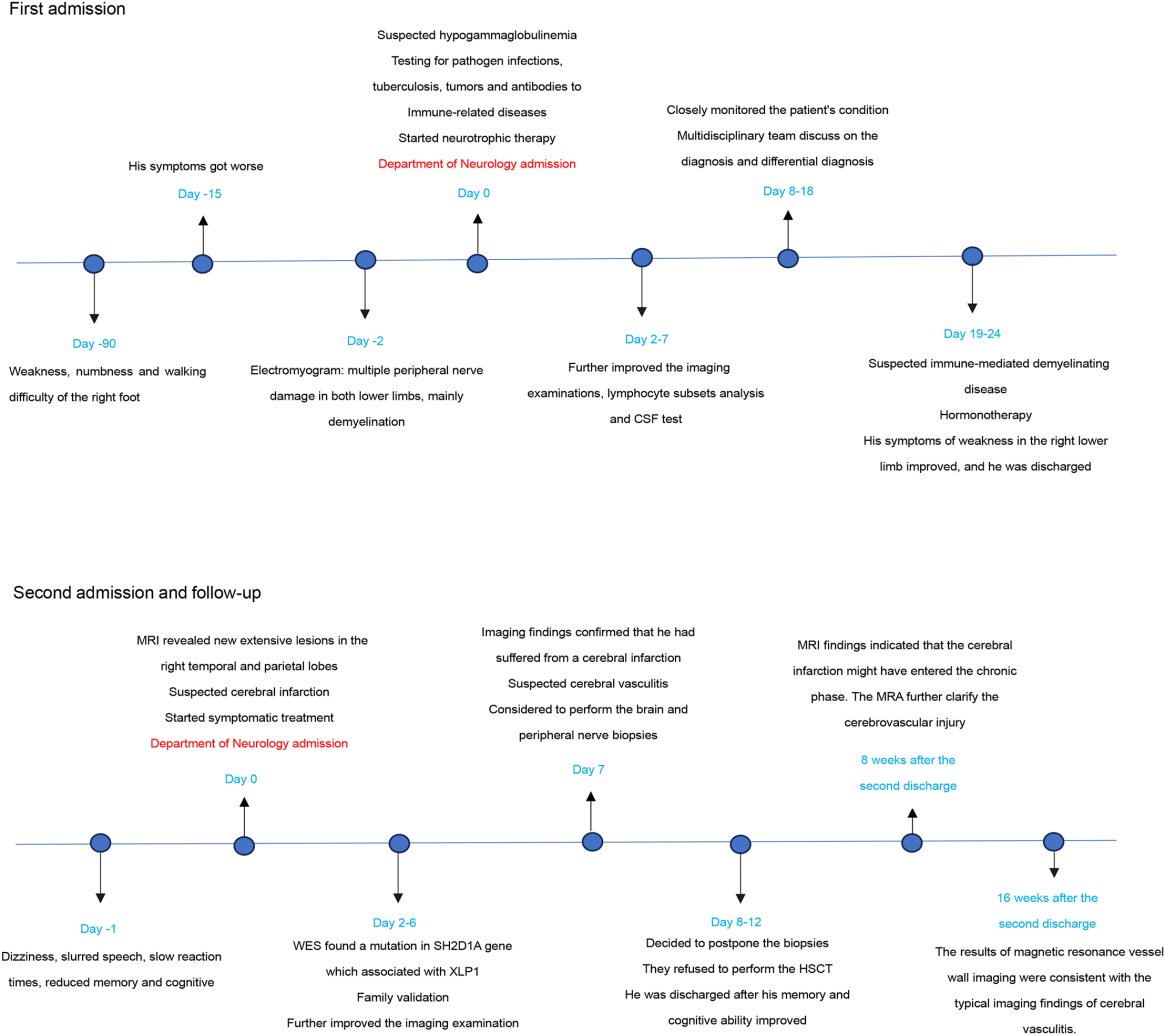
Timeline of the case report.

MRI findings obtained 8 weeks after the discharge revealed multiple encephalomalacia foci with high intensities on T2-weighted images in the right temporal and parietal lobes (**Figure 2A**). The DWI image presented no limited diffusion (**Figure 2B**), the ADC image presented high intensities in the right temporal and parietal lobes (**Figure 2C**), which indicated that the cerebral infarction might have entered the chronic phase. MRA revealed that anterior, middle, and posterior cerebral arteries and their branches were rough and stiff on both sides, the right middle cerebral artery was thinner than that of the left, with fewer branches (**Figure 2D**). MRA findings obtained 16 weeks after the discharge showed a thickened vascular shadow beside the M1 bifurcation of the right middle cerebral artery, its distal end and branches were not visualized (**Figure 2E**). Magnetic resonance vessel wall imaging showed homogeneous concentric thickening with enhancement in the M1 segment of the right middle cerebral artery (**Figures 2F-L**), which was consistent with the typical imaging findings of cerebral vasculitis. The timeline of case report was shown in **Figure 3**. We will continue to monitor the patient’s condition and provide further updates.

## Discussion

In this case, the main clinical manifestations of the XLP1 patient during his two admissions included multiple peripheral nerve demyelinating lesions and cerebrovascular injury which resulted in cerebral infarction, but no EBV infection in both two admissions, which was a rare occurrence. WES confirmed the c.163C>T mutation of *SH2D1A* gene as the pathogenic mutation site. Five patients with the same mutation have been reported previously (5–7). Among them, two Chinese boys from the same family (aged 9 and 16 respectively) presented with agammaglobulinemia, recurrent respiratory infections and EBV infection. Interestingly, the other three patients (aged 9, 22 and 31 respectively) both presented with CNS vasculitis (two of them were non-EBV associated CNS vasculitis, the other one tested positive for CSF EBV but negative for blood EBV). Combined with the condition in our case, we speculated that this mutation might be associated with the occurrence of CNS vasculitis, especially in the absence of EBV. Or other, patients with this mutation may have a greater tendency to develop CNS vasculitis with age. It also indicates that the clinical heterogeneity between genotypes and phenotypes of XLP1 needs to be considered in terms of specific mutations.

Imaging findings confirmed that the patient had suffered from a cerebral infarction during his second admission. As for etiology, according to the TOAST (Trial of Org 10172 in Acute Stroke Treatment) system, which was composed of five major subtypes: large artery atherosclerosis (LAA, embolus/thrombosis), cardioembolism (CE, high-risk/medium-risk), small artery occlusion (SAO, defined by the clinical syndrome and the diameter of the infarct ≤ 15 mm), stroke of other determined cause (SOC), and stroke of undetermined cause (SUC). SUC was further divided into (1) no cause was found despite an extensive workup or (2) two or more plausible causes were found (8). Based on the patient’s medical history, laboratory tests and imaging findings, his cerebral infarction was due to stroke of other determined cause (SOC). Further analyzed the patient’s MRI findings, multi-infarct lesions involved the cerebral cortex with high intensities on T2-Weight images, especially the magnetic resonance vessel wall imaging showed homogeneous concentric thickening with enhancement in the M1 segment of the right middle cerebral artery. These features were consistent with the typical imaging findings of cerebral vasculitis, which have been reported in pathologically confirmed patients with cerebral vasculitis (9), although it is an extremely rare complication of XLP1. cerebral vasculitis has been reported in only 13 patients with XLP in the past 20 years (10). If his condition has other changes in the future, we will consider a biopsy to confirm our conclusion and provide the results if necessary.

The disease best matched the patient’s peripheral nerve demyelinating lesions is chronic inflammatory demyelinating multiple radiculopathy (CIDP). CIDP is a type of immune-mediated demyelinating peripheral neuropathy, the most common clinical manifestation is multiple motor sensory peripheral neuropathy. It can be divided into classical CIDP and variant CIDP. Neuroelectrophysiological examination is necessary for the diagnosis of CIDP, which can be used to provide evidence of demyelinating lesions. In addition, the progression time of CIDP from the onset of symptoms to the most severe stage of the disease is at least 8 weeks, and the phenomenon of protein separation in cerebrospinal fluid can be one of the conditions to support the diagnosis of CIDP (11, 12). The process of this patient from the initial detection of lower limb weakness to the exacerbation of symptoms lasted for more than 2 mouths, and electromyogram findings confirmed the presence of demyelinating lesions in more than 2 nerves. The above 2 points have met the clinical and electrophysiological criteria for the diagnosis of CIDP. In addition, with no evidence of cerebral hemorrhage, tumors or infections, the patient’s two CSF tests only showed a significant increase in protein, which belonged to a phenomenon of CSF-protein and cells separation that may occur in CIDP, and his clinical manifestations were closest to variant CIDP. As with the cerebrovascular injury, we believed that the patient’s peripheral nerve demyelinating lesions could be attributed to the immune-mediated accident which possibly caused by a lymphocytic abnormal proliferation disorder associated with XLP1, but a peripheral nerve biopsy are unavailable for the time being, and we have stated the reasons.

HLH is a common clinical manifestation of XLP, usually occurs after EBV infection, and which is a heterogeneous life-threatening inflammatory response disease (3). The incidence of HLH involvement in the CNS ranges from 30% to 73%, and the predisposing factors of CNS-HLH include infection (accounting for more than 50%), malignant tumors, and autoimmune diseases. CNS-HLH often caused bilateral lesions, with typical MRI findings of abnormal white matter signals around the ventricles, accompanied by volume loss and ventricular enlargement. meningeal and perivascular enhancement are commonly seen (13, 14). In our case, there was no evidence of systemic HLH with him. In addition, Imaging findings were more consistent with typical cerebral vasculitis rather than CNS-HLH. A brain biopsy will further clarify the pathological features as circumstances dictate.

When conducting multidisciplinary discussions, we have considered whether the patient met the diagnosis of multiple sclerosis (MS) and ultimately excluded the diagnosis. The diagnosis of MS can only be established with clinical and/or radiological demonstration of lesions in the CNS that are disseminated in space (DIS) and time (DIT). MRI is the most sensitive tool to detect the presence of brain and spinal cord lesions in MS (15). According to the 2010 McDonald criteria and new 2017 McDonald criteria and the patient’s multiple MRI results (more than 5 times) from admission to follow-up after discharge, the DIS/DIT criteria can’t be met. In addition, there were no MS-specific findings in these MRI results such as Dawson fingers sign. In terms of disease progression, the patient did not meet any of the four clinical phenotypes of multiple sclerosis (relapsing-remitting, secondary-progressive, primary progressive, and progressive relapsing). Laboratory testing showed no clear viral infection, negative for multiple autoimmune disease-associated antibodies (we did not test for CSF-specific oligoclonal bands because this examination must be established on meeting the DIS criteria). He also had no family history of MS. Based on the above results, we believe that the diagnosis of MS can be ruled out.

HSCT is an effective treatment for XLP1. Among the reported 13 cases of XLP1-related cerebral vasculitis, 10 patients did not undergo HSCT, 9 have died, showing a poor outcome of cerebral vasculitis in XLP1. 3 patients underwent HSCT, with two improving and one dying. Among the reported 5 cases with the same mutation as this patient, 4 patients did not undergo HSCT, with 2 dying. 1 patient underwent HSCT, showing clinical improvement. In our case, the patient developed cerebral vasculitis and peripheral nerve demyelinating lesions but no life-threatening HLH, and HSCT might improve his prognosis. It was a pity that they refused the HSCT treatment for some special reasons.

This is the first case reported multiple peripheral nerve demyelinating lesions appeared in a XLP1 patient without evidence of EBV infection or other infections, which is a rare occurrence and expands our understanding of XLP1 complications. Besides, the case shows a potential to study on the immune-related mechanism of possibly lymphocytic abnormal proliferation disorder associated with XLP1 involving both peripheral nerves and cerebrovascular. Last but not the least, this is an ongoing and dynamic study, we’ll continue to record his condition to provide a valuable reference of other XLP1 patients’ assessment on their disease progression, prognosis and outcomes whose clinical findings are similar to this case and without undergoing HSCT.

We were unable to perform the brain and peripheral nerve biopsies because of the potential adverse effect on the patient, which we’ll consider to perform and provide the results if his condition has other changes in the future.

There are some lessons to be drawn from this case. For some XLP1 patients, their clinical manifestations are atypical, and diagnostic vigilance should be raised. When any potential immune deficiency manifestations of them were revealed, genetic testing should be performed as early as possible. For patients diagnosed with XLP1 but not undergoing a HSCT, their conditions, especially the central and peripheral nervous systems, should be closely monitored in order to detect early lesions and prevent serious consequences. In addition, necessary genetic screening, fertility counseling, and prenatal diagnosis should be conducted for other family members of XLP1 patients.

## Data Availability

The datasets presented in this article are not readily available due to ethical and privacy-related concerns. Requests to access the datasets should be directed to the corresponding author.

## Supplementary material

Supplementary Figure 1
**(A)** Whole exome sequencing map of the family line. The patient had a hemizygous mutation in *SH2D1A* gene (c.163C>T: p.R55*), a heterozygous mutation in his mother, no mutation in his father, and the mutation came from his mother. **(B)** Sanger sequencing of the *SH2D1A* gene in patient’s maternal family members. **(C)** The patient’s maternal genealogical tables. **(D)** Wild-type SAP protein structure. (E) Structural prediction of mutant SAP protein. The *SH2D1A* gene mutation in this patient, family-based validation of the mutation site, the patient’s genealogical tables and structural prediction of mutant SAP protein.

Supplementary Table 1 Detailed laboratory test results of the patient.

Supplementary Table 2 The variation information of the patient and his parents.
