# Supplementary figures and images for: Case Report: Multiple peripheral nerve demyelinating lesions and cerebrovascular injury which resulted in extensive cerebral infarction in a XLP1 patient without EBV infection
Source: Front Immunol. 2025 May 30;16:1580909. doi: 10.3389/fimmu.2025.1580909 (PMC12162628; doi:10.3389/fimmu.2025.1580909)

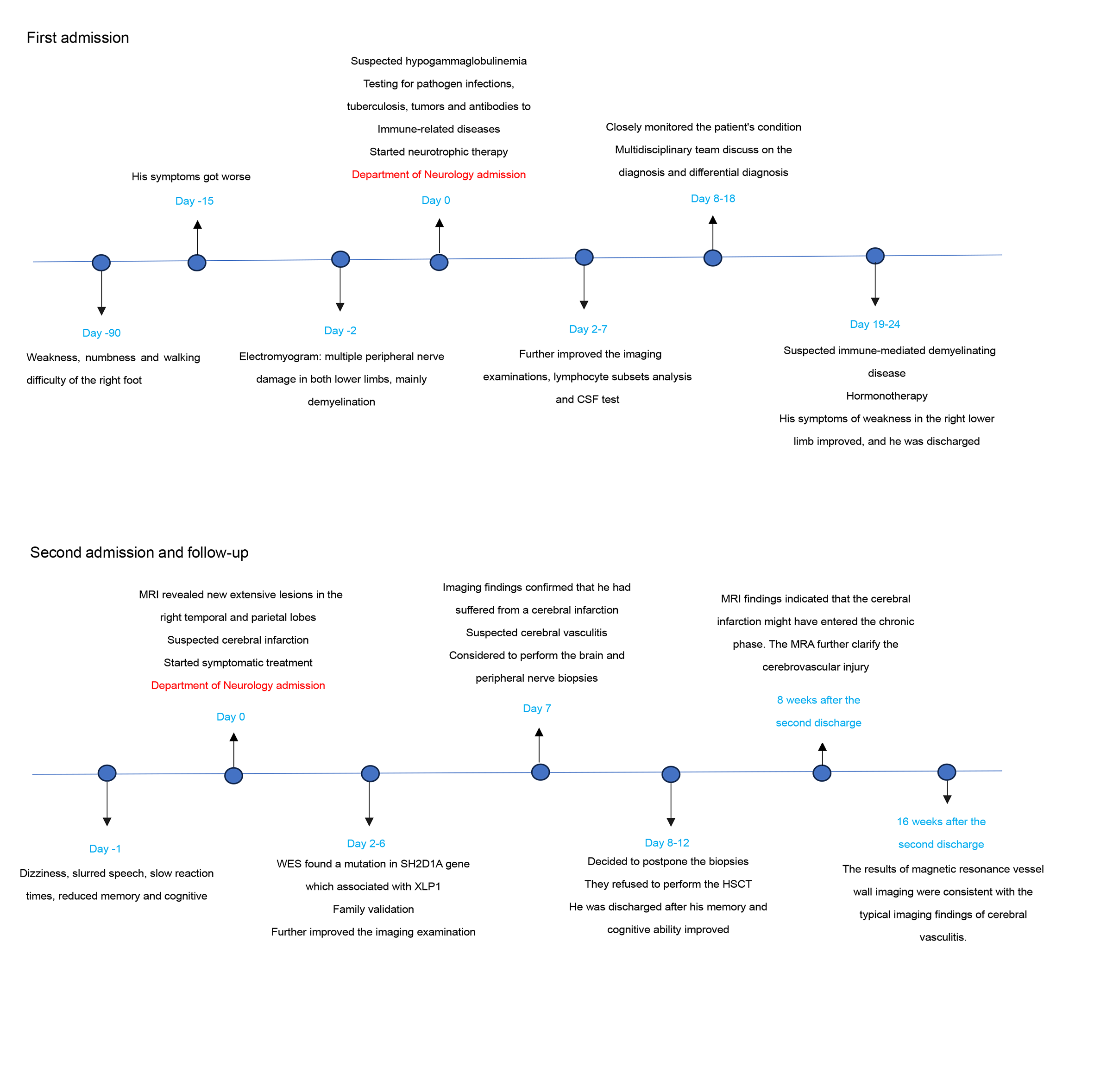

Supplement: Supplementary Figure 1 — (A) Whole exome sequencing map of the family line. The patient had a hemizygous mutation in SH2D1A gene (c.163C>T: p.R55*), a heterozygous mutation in his mother, no mutation in his father, and the mutation came from his mother. (B) Sanger sequencing of the SH2D1A gene in patient’s maternal family members. (C) The patient’s maternal genealogical tables. (D) Wild-type SAP protein structure. (E) Structural prediction of mutant SAP protein. The SH2D1A gene mutation in this patient, family-based validation of the mutation site, the patient’s genealogical tables and structural prediction of mutant SAP protein. [file Image1.tif]

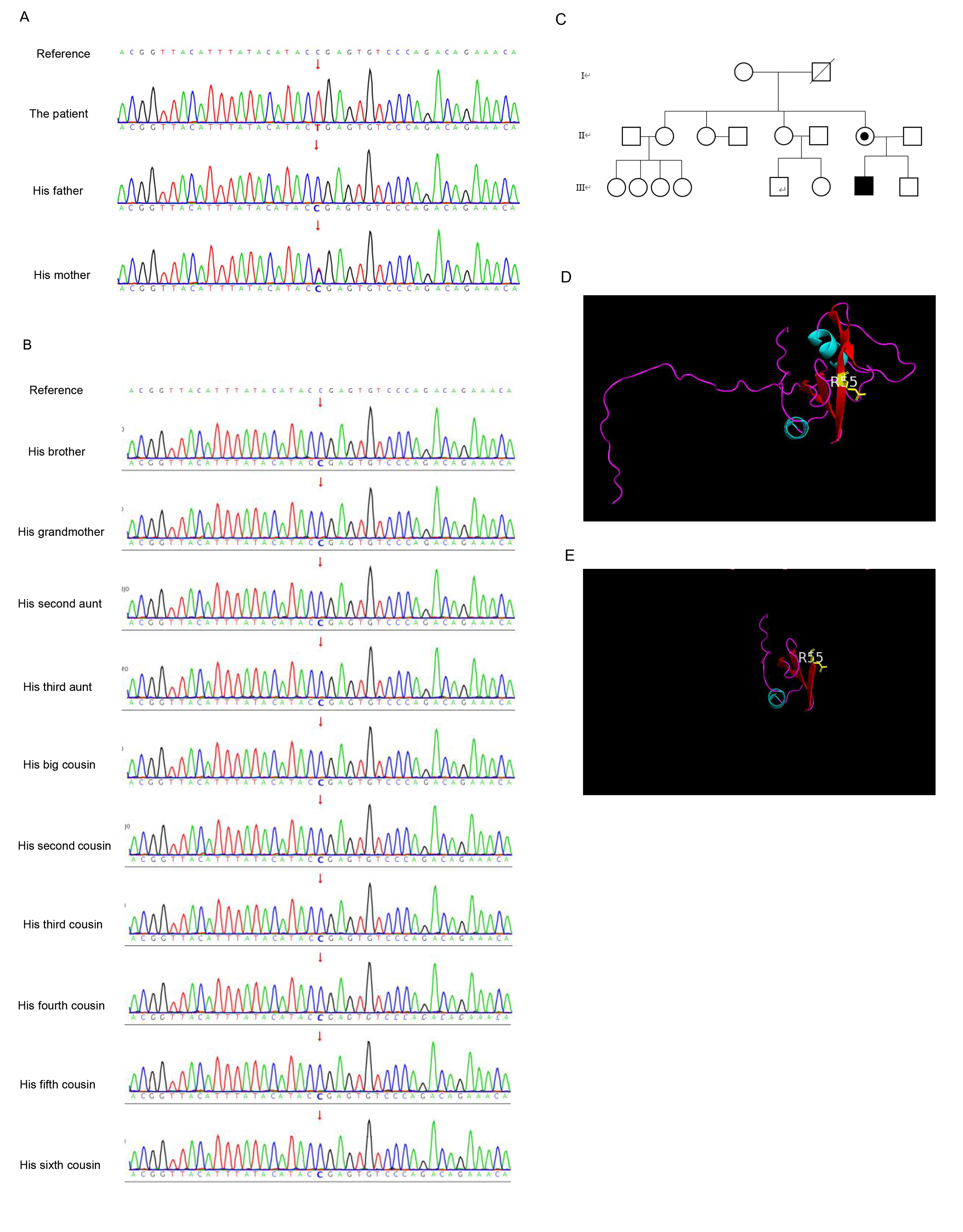

Supplement: Supplementary file 2 [file Image2.tif]
