# Supplementary material for: Case Report: Multiple peripheral nerve demyelinating lesions and cerebrovascular injury which resulted in extensive cerebral infarction in a XLP1 patient without EBV infection
Source: Front Immunol. 2025 May 30;16:1580909. doi: 10.3389/fimmu.2025.1580909 (PMC12162628; doi:10.3389/fimmu.2025.1580909)
Supplement: Supplementary Table 1 — Detailed laboratory test results of the patient. [file Table1.docx]

Results of laboratory tests two days before the patient's first admission

| Laboratory indicators | Case (16yrs) | Normal range (age-matched) |
| --- | --- | --- |
| White blood cell (WBC), ×10^9^/L | 8.46 | 4.1-11.0 |
| Red blood cell (RBC), ×10^9^/L | 5.75 | 4.5-5.9 |
| Hemoglobin (HGB), g/L | 149.80 | 129.00-172.00 |
| Platelet (PLT), ×10^9^/L | 233.40 | 150.00-407.00 |
| Neutrophil (%) | 0.42 | 0.37-0.77 |
| Lymphocyte (%) | 0.48 | 0.17-0.54 |
| Monocyte (%) | 0.06 | 0.02-0.11 |
| Neutrophil, ×10^9^/L | 3.57 | 1.8-8.3 |
| Lymphocyte, ×10^9^/L | 4.04↑ | 1.2-3.8 |
| Monocyte, ×10^9^/L | 0.48 | 0.14-0.74 |
| Eosinophil (%) | 0.034 | 0.00-0.09 |
| Basophil (%) | 0.007 | 0.00-0.01 |
| Eosinophil, ×10^9^/L | 0.29 | 0.00-0.68 |
| Basophil, ×10^9^/L | 0.08↑ | 0.00-0.07 |
| Mean corpuscular volume (MCV), fl | 80.35 | 80-100 |
| Mean corpuscular hemoglobin (MCH), pg | 26.07 | 25-34 |
| Mean corpuscular hemoglobin concentration (MCHC), g/L | 324.40 | 310-355 |
| Hematocrit value (HCT) | 0.46 | 0.39-0.51 |
| Platelet hematocrit (PCT) | 0.19 | 0.11-0.27 |
| Total bilirubin (TBil), umol/L | 11.8 | 0-26 |
| Direct bilirubin (DBil), umol/L | 2.1 | 0-6.8 |
| Indirect bilirubin (IBil), umol/L | 9.7 | 3.1-14.3 |
| Aspartate aminotransferase (AST), U/L | 19 | 12-37 |
| Alanine aminotransferase (ALT), U/L | 9 | 7-43 |
| Creatine kinase (CK), U/L | 139 | 50-310 |
| CK-MB, U/L | 23 | 0-25 |
| Lactate dehydrogenase (LD), U/L | 197 | 120-250 |
| Lactic acid, mmol/L | 2.500↑ | 0.600-2.200 |
| Total Protein, g/L | 72.7 | 68.0-88.0 |
| Albumin, g/L | 44.4 | 42.0-56.0 |
| Globulin, g/L | 28.3 | 19.0-40.0 |
| Total bile acid, umol/L | 5.30 | 0.00-10.00 |
| Alkaline phosphatase (ALP), U/L | 207 | 64-443 |
| Blood kalium, mmol/L | 3.83 | 3.50-4.90 |
| Blood natrium, mmol/L | 138.70 | 135.00-145.00 |
| Blood chlorine, mmol/L | 105.60 | 98.00-110.00 |
| Blood calcium (Total), mmol/L | 2.40 | 2.10-2.80 |
| Blood magnesium, mmol/L | 0.82 | 0.50-0.90 |
| Blood phosphorus, mmol/L | 1.13 | 0.84-1.71 |

Results of laboratory tests on the day of his first admission

| Laboratory indicators | Case (16yrs) | Normal range (age-matched) |
| --- | --- | --- |
| White blood cell (WBC), ×109/L | 8.52 | 4.1-11.0 |
| Red blood cell (RBC), ×109/L | 5.35 | 4.5-5.9 |
| Hemoglobin (HGB), g/L | 140.20 | 129.00-172.00 |
| Platelet (PLT), ×109/L | 205.00 | 150.00-407.00 |
| Neutrophil (%) | 0.42 | 0.37-0.77 |
| Lymphocyte (%) | 0.44 | 0.17-0.54 |
| Monocyte (%) | 0.08 | 0.02-0.11 |
| Neutrophil, ×109/L | 3.61 | 1.8-8.3 |
| Lymphocyte, ×109/L | 3.72 | 1.2-3.8 |
| Monocyte, ×109/L | 0.64 | 0.14-0.74 |
| Eosinophil (%) | 0.05 | 0.00-0.09 |
| Basophil (%) | 0.009 | 0.00-0.01 |
| Eosinophil, ×109/L | 0.47 | 0.00-0.68 |
| Basophil, ×109/L | 0.07 | 0.00-0.07 |
| Mean corpuscular volume (MCV), fl | 77.84↓ | 80-100 |
| Mean corpuscular hemoglobin (MCH), pg | 26.19 | 25-34 |
| Mean corpuscular hemoglobin concentration (MCHC), g/L | 336.50 | 310-355 |
| Hematocrit value (HCT) | 0.42 | 0.39-0.51 |
| Platelet hematocrit (PCT) | 0.18 | 0.11-0.27 |
| Total bilirubin (TBil), umol/L | 13.7 | 0-26 |
| Direct bilirubin (DBil), umol/L | 3.0 | 0-6.8 |
| Indirect bilirubin (IBil), umol/L | 10.7 | 3.1-14.3 |
| Aspartate aminotransferase (AST), U/L | 24 | 12-37 |
| Alanine aminotransferase (ALT), U/L | 17 | 7-43 |
| Creatine kinase (CK), U/L | 133 | 50-310 |
| CK-MB, U/L | 23 | 0-25 |
| Lactate dehydrogenase (LD), U/L | 175 | 120-250 |
| Lactic acid, mmol/L | 2.62↑ | 0.600-2.200 |
| Total Protein, g/L | 66.1↓ | 68-88 |
| Prealbumin, mg/L | 117.9↓ | 200-430 |
| Albumin, g/L | 38.6↓ | 42-56 |
| Globulin, g/L | 27.5 | 19-40 |
| Urea nitrogen, mmol/L | 3.03 | 2.70-7.70 |
| Serum creatinine, umol/L | 68 | 52-101 |
| Uric acid, umol/L | 297 | 208-428 |
| Bicarbonate radical, mmol/L | 22.80 | 22.00-29.00 |
| Endogenous creatinine clearance rate, ml/min | 83.0↓ | 85.0-125.0 |
| Cystatin C, mg/L | 0.92 | 0.59-1.03 |
| C-reactive protein (CRP), mg/L | 36.69↑ | 0-10 |
| Interleukin-6 (IL-6), pg/ml | 2.10 | 0-7 |
| Ferritin, ng/ml | 170.64 | 21.80-274.66 |
| Ferrum, umol/L | 4.90↓ | 9.0-32.2 |
| Total iron binding capacity, umol/L | 48.20↓ | 50-77 |
| Iron saturation, umol/L | 0.10↓ | 0.28-0.50 |
| Unsaturated iron binding capacity, umol/L | 43.30 | 30.00-54.00 |
| Alpha-hydroxybutyrate dehydrogenase  (α-HBD), U/L | 115 | 72-182 |
| Immunoglobulin G (IgG), g/L | 11.44 | 8.6-17.4 |
| Immunoglobulin A (IgA), g/L | 0.01↓ | 1.0-4.2 |
| Immunoglobulin M (IgM), g/L | 2.63↑ | 0.3-2.2 |
| Immunoglobulin G4 (IgG4), g/L | 0.01 | 0.00-2.00 |
| Complement C3, g/L | 1.53↑ | 0.70-1.40 |
| Complement C4, g/L | 0.78↑ | 0.10-0.40 |
| Antistreptolysin O (ASO), IU/ml | 23 | ≤170 |
| Rheumatoid factor (RF), IU/ml | 21.0↑ | ≤18.0 |
| HBsAg, IU/ml | 0.00 | 0.00-0.05 |
| Anti-HBs, mIU/ml | 0.04 | 0.00-10.00 |
| HBeAg, PElu/ml | 0.00 | 0.00-0.50 |
| Anti-HBe, PElu/ml | 0.01 | 0.00-0.20 |
| Anti-HBc, PElu/ml | 0.78 | 0.00-0.90 |
| Hepatitis C virus (HCV), S/CO | 0.03 | 0.00-1.00 |
| Human immunodeficiency virus (HIV), S/CO | 0.17 | 0.00-1.00 |
| Treponema pallidum, S/CO | 0.05 | 0.00-1.00 |
| Influenza A virus, S/CO | negative | negative |
| Influenza B virus, S/CO | negative | negative |
| Epstein-Barr virus VCA-IgA | negative | negative |
| Epstein-Barr virus nuclear antigen IgA | negative | negative |
| Epstein-Barr virus VCA-IgG | negative | negative |
| Epstein-Barr virus nuclear antigen IgG | negative | negative |
| Epstein-Barr virus PCR | negative | negative |
| Herpes simplex virus 1/2 PCR | negative | negative |
| Cytomegalovirus PCR | negative | negative |
| Prothrombin Time (PT), s | 13.00 | 9-15 |
| Prothrombin activity | 1.17 | 0.8-1.4 |
| Thrombin time (TT), s | 13.00 | 9-15 |
| Activated Partial Thromboplastin Time (APTT), s | 35.80 | 23-40 |
| Fibrinogen (%) | 78 | 70-130 |
| International Normalised Ratio (INR) | 1.17 | 0.8-1.4 |
| Total cholesterol, mmol/L | 3.73 | <5.20 |
| Triglyceride, mmol/L | 0.90 | <1.70 |
| High-density lipoprotein, mmol/L | 1.03 | ≥1.00 |
| Low-density lipoprotein, mmol/L | 2.30 | <3.40 |
| Blood glucose, mmol/L | 4.31 | 3.9-6.1 |
| Folate, ng/ml | 4.40 | >4.00 |
| Vitamin B12, pg/ml | 418.00 | 187-883 |
| D-dimer, ng/ml | 157 | 0-450 |
| Blood kalium, mmol/L | 3.70 | 3.50-4.90 |
| Blood natrium, mmol/L | 136.40 | 135.00-145.00 |
| Blood chlorine, mmol/L | 103.60 | 98.00-110.00 |
| Blood calcium (Total), mmol/L | 2.31 | 2.10-2.80 |
| Blood magnesium, mmol/L | 0.82 | 0.50-0.90 |
| Blood phosphorus, mmol/L | 1.28 | 0.84-1.71 |
| Blood cuprum, umol/L | 22.90 | 10.99-21.98 |
| Blood zinc, umol/L | 10.50 | 9.00-20.70 |
| Alkaline phosphatase (ALP), U/L | 130 | 64-443 |
| Cholinesterase, U/L | 7615 | 5000-20000 |
| Total bile acid, umol/L | 6.7 | 0-10 |
| Alpha-fetoprotein (AFP), ng/ml | 0.95 | ≤7.00 |
| Carcinoembryonic antigen (CEA), ng/ml | 1.94 | 0.00-5.00 |
| CA125, U/L | 21.50 | 0.00-35.00 |
| CA153, U/L | 14.80 | 0.00-31.00 |
| CA199, U/L | <2.00 | 0.00-37.00 |
| Troponin, ng/ml | 0.0010 | 0.0000-0.0297 |
| CYFRA21-1, ng/ml | 1.28 | 0.00-3.30 |
| Squamous cell carcinoma antigen (SCCA), ng/ml | 1.20 | 0.00-1.50 |
| Anti-cyclic citrulline polypeptide antibody, U/L | 0.40 | 0.00-5.00 |
| Vitamin D3, nmol/L | 33.30 | 0-10 |
| Vitamin D2, nmol/L | 3.50 | 5000-20000 |
| Vitamin D, nmol/L | 36.80↓ | 50.00-250.00 |

Test results of anti-nuclear antibody + anti-nuclear extract antibody + anti-neutrophil cytoplasmic antibody + anti-cardiolipin antibody on the day of his first admission

| Laboratory indicators | Case (16yrs) | Normal range (age-matched) |
| --- | --- | --- |
| ANA-llF | negative | negative |
| dsDNA-llF | negative | negative |
| ACA-llF | negative | negative |
| nRNP/Sm-LIA | negative | negative |
| Sm-LIA | negative | negative |
| SSA-LIA | negative | negative |
| Ro52-LIA | negative | negative |
| SSB-LIA | negative | negative |
| Scl70-LIA | negative | negative |
| Jo1-LIA | negative | negative |
| CENPB-LIA | negative | negative |
| dsDNA-LIA | negative | negative |
| Nuk-LIA | negative | negative |
| His-LIA | negative | negative |
| rRNP-LIA | negative | negative |
| pANCA-llF | negative | negative |
| cANCA-llF | negative | negative |
| aANCA-llF | negative | negative |
| MPO IgG, AU/ml | 1.6 | <20.00 |
| ACL IgG, GPLU/ml | 1.00 | <10.00 |
| ACL IgM, MPLU/ml | 2.50 | <10.00 |
| ACL IgA, APLU/ml | <2.50 | <10.00 |

Results of laboratory tests on the 7th day of his first admission

| Laboratory indicators | Case (16yrs) | Normal range (age-matched) |
| --- | --- | --- |
| White blood cell (WBC), ×109/L | 8.58 | 4.1-11.0 |
| Red blood cell (RBC), ×109/L | 5.06 | 4.5-5.9 |
| Hemoglobin (HGB), g/L | 129.30 | 129.00-172.00 |
| Platelet (PLT), ×109/L | 433.10↑ | 150.00-407.00 |
| Neutrophil (%) | 0.533 | 0.37-0.77 |
| Lymphocyte (%) | 0.29 | 0.17-0.54 |
| Monocyte (%) | 0.06 | 0.02-0.11 |
| Neutrophil, ×109/L | 4.57 | 1.8-8.3 |
| Lymphocyte, ×109/L | 2.45 | 1.2-3.8 |
| Monocyte, ×109/L | 0.49 | 0.14-0.74 |
| Eosinophil (%) | 0.1123↑ | 0.00-0.09 |
| Basophil (%) | 0.0124↑ | 0.00-0.01 |
| Eosinophil, ×109/L | 0.964↑ | 0.00-0.68 |
| Basophil, ×109/L | 0.11↑ | 0.00-0.07 |
| Mean corpuscular volume (MCV), fl | 77.30↓ | 80-100 |
| Mean corpuscular hemoglobin (MCH), pg | 25.58 | 25-34 |
| Mean corpuscular hemoglobin concentration (MCHC), g/L | 330.90 | 310-355 |
| Hematocrit value (HCT) | 0.391 | 0.39-0.51 |
| Platelet hematocrit (PCT) | 0.34↑ | 0.11-0.27 |
| Urea nitrogen, mmol/L | 1.81↓ | 2.70-7.70 |
| Serum creatinine, umol/L | 54 | 52-101 |
| Uric acid, umol/L | 182↓ | 208-428 |
| Aspartate aminotransferase (AST), U/L | 23 | 12-37 |
| Alanine aminotransferase (ALT), U/L | 29 | 7-43 |
| Immunoglobulin G (IgG), g/L | 9.49 | 8.6-17.4 |
| Immunoglobulin A (IgA), g/L | 0.01↓ | 1.0-4.2 |
| Immunoglobulin M (IgM), g/L | 2.21↑ | 0.3-2.2 |
| Immunoglobulin G4 (IgG4), g/L | 0.03 | 0.00-2.00 |
| Erythrocyte sedimentation rate (ESR), mm | 40↑ | 0-15 |
| Total protein, g/L | 66.1↓ | 68-88 |
| Albumin, g/L | 38.6↓ | 42-56 |
| Globulin, g/L | 27.5 | 19-40 |
| Procalcitonin (PCT), ng/ml | 0.051↑ |  |
| Mycoplasma pneumoniae antibody quantification | <1:40 | 1:40 |
| GM test | 0.124 | <0.5 |

Results of cerebrospinal fluid (CSF) examination on the 7th day of his first admission

| Laboratory indicators | Case (16yrs) | Normal range (age-matched) |
| --- | --- | --- |
| Color | Bright yellow |  |
| CSF-pressure, mmH_2_O | 95 | 80-180 |
| Pandy's test | 2+ | negative |
| Total number of nucleated cells, ×10^6^/L | 12↑ | 0-10 |
| CSF-GLU, mmol/L | 2.49↓ | 2.50-4.50 |
| CSF-Chloride, mmol/L | 116.90↓ | 120.00-132.00 |
| CSF-Protein, mg/L | 4563.10↑ | 150.00-450.00 |
| CSF-ADA, U/L | 8.70↑ | 0.00-8.00 |
| CSF-CK, U/L | 14 | 0-30 |
| CSF-LD, U/L | 219↑ | 3-40 |
| CSF-ALT, U/L | 152↑ | 5-20 |
| CSF-Ca, mmol/L | 1.23 | 1.00-1.40 |
| CSF-Mg, mmol/L | 1.02↓ | 1.10-1.40 |
| CSF-Alb, mg/L | 4088.00 |  |
| CSF-IgG, mg/L | 903.60↑ | 10.00-40.00 |
| CSF-NGS | negative | negative |
| CSF-Xpert | negative | negative |
| CSF-GFAP | negative | negative |

Results of lymphocyte subsets on the 7th day of his first admission

| Laboratory indicators | Case (16yrs) | Normal range (age-matched) |
| --- | --- | --- |
| Total T cells | 89.78%↑ | 62.6%-76.8% |
| CD4+ | 39.51% | 30%-46% |
| CD4+ (absolute count) | 1037 | 561-1137 |
| CD8+ | 42.53%↑ | 19.2%-33.6% |
| CD8+ (absolute count) | 1116↑ | 220-1030 |
| CD4+ / CD8+ | 0.93↓ | 0.95-2.13 |
| CD19+ | 7.41%↓ | 8.5%-14.5% |
| NK cells | 2.30%↓ | 9.5%-23.5% |
| CD3+CD4+CD8+ | 1.08% |  |
| CD3+CD4-CD8- | 8.82% |  |
| CD3-CD19+ (absolute count) | 201 | 180-324 |
| CD16+CD56+ (absolute count) | 62↓ | 200-567 |

Results of laboratory tests on the 15th day of his first admission

| Laboratory indicators | Case (16yrs) | Normal range (age-matched) |
| --- | --- | --- |
| White blood cell (WBC), ×109/L | 9.47 | 4.1-11.0 |
| Red blood cell (RBC), ×109/L | 5.48 | 4.5-5.9 |
| Hemoglobin (HGB), g/L | 142.3 | 129.00-172.00 |
| Platelet (PLT), ×109/L | 506.2↑ | 150.00-407.00 |
| Neutrophil (%) | 0.047 | 0.37-0.77 |
| Lymphocyte (%) | 0.040 | 0.17-0.54 |
| Monocyte (%) | 0.044 | 0.02-0.11 |
| Neutrophil, ×109/L | 4.44 | 1.8-8.3 |
| Lymphocyte, ×109/L | 3.81↑ | 1.2-3.8 |
| Monocyte, ×109/L | 0.42 | 0.14-0.74 |
| Eosinophil (%) | 0.067↑ | 0.00-0.09 |
| Basophil (%) | 0.018↑ | 0.00-0.01 |
| Eosinophil, ×109/L | 0.64 | 0.00-0.68 |
| Basophil, ×109/L | 0.17↑ | 0.00-0.07 |
| Mean corpuscular volume (MCV), fl | 78.50↓ | 80-100 |
| Mean corpuscular hemoglobin (MCH), pg | 25.95 | 25-34 |
| Mean corpuscular hemoglobin concentration (MCHC), g/L | 330.60 | 310-355 |
| Hematocrit value (HCT) | 0.430 | 0.39-0.51 |
| Platelet hematocrit (PCT) | 0.39↑ | 0.11-0.27 |
| Urea nitrogen, mmol/L | 3.74 | 2.70-7.70 |
| Serum creatinine, umol/L | 48↓ | 52-101 |
| Uric acid, umol/L | 189↓ | 208-428 |
| Aspartate aminotransferase (AST), U/L | 28 | 12-37 |
| Alanine aminotransferase (ALT), U/L | 30 | 7-43 |
| C-reactive protein (CRP), mg/L | <10.00 | 0-10 |
| Erythrocyte sedimentation rate (ESR), mm | 22↑ | 0-15 |
| Procalcitonin (PCT), ng/ml | 0.033 |  |
| Total protein, g/L | 66.1↓ | 68-88 |
| Albumin, g/L | 38.6↓ | 42-56 |
| Globulin, g/L | 27.5 | 19-40 |
| Epstein-Barr virus PCR | negative | negative |
| Epstein-Barr virus VCA-IgG | negative | negative |
| Epstein-Barr virus nuclear antigen IgG | negative | negative |

Serum immunofixation electrophoresis results of the patient on the 15th day of his first admission

| Laboratory indicators | Case (16yrs) | Normal range (age-matched) |
| --- | --- | --- |
| IgG | negative | negative |
| IgA | negative | negative |
| IgM | negative | negative |
| k Light chain | negative | negative |
| λ Light chain | negative | negative |

Results of cerebrospinal fluid (CSF) reexamination on the 21st day of his first admission

| Laboratory indicators | Case (16yrs) | Normal range (age-matched) |
| --- | --- | --- |
| Color | Clear light yellow |  |
| CSF-pressure, mmH_2_O | 95 | 80-180 |
| Pandy's test | 2+ | negative |
| Total number of nucleated cells, ×10^6^/L | 15↑ | 0-10 |
| CSF-GLU, mmol/L | 2.18↓ | 2.50-4.50 |
| CSF-Chloride, mmol/L | 127.80 | 120.00-132.00 |
| CSF-Protein, mg/L | 4467.50↑ | 150.00-450.00 |
| CSF-ADA, U/L | 9.00↑ | 0.00-8.00 |
| CSF-CK, U/L | 16 | 0-30 |
| CSF-LD, U/L | 218↑ | 3-40 |
| CSF-ALT, U/L | 143↑ | 5-20 |
| CSF-Ca, mmol/L | 1.40 | 1.00-1.40 |
| CSF-Mg, mmol/L | 1.11 | 1.10-1.40 |
| CSF-Alb, mg/L | 4152.90 |  |
| CSF-IgG, mg/L | 1104.90↑ | 10.00-40.00 |
| CSF-NGS | negative | negative |
| CSF-Xpert | negative | negative |
| CSF-GFAP | negative | negative |

Results of laboratory tests prior to his second admission

| Laboratory indicators | Case (16yrs) | Normal range (age-matched) |
| --- | --- | --- |
| White blood cell (WBC), ×10^9^/L | 7.16 | 4.1-11.0 |
| Red blood cell (RBC), ×10^9^/L | 5.42 | 4.5-5.9 |
| Hemoglobin (HGB), g/L | 145.30 | 129.00-172.00 |
| Platelet (PLT), ×10^9^/L | 207.90 | 150.00-407.00 |
| Neutrophil (%) | 0.494 | 0.37-0.77 |
| Lymphocyte (%) | 0.373 | 0.17-0.54 |
| Monocyte (%) | 0.06 | 0.02-0.11 |
| Neutrophil, ×10^9^/L | 3.54 | 1.8-8.3 |
| Lymphocyte, ×10^9^/L | 2.68 | 1.2-3.8 |
| Monocyte, ×10^9^/L | 0.43 | 0.14-0.74 |
| Eosinophil (%) | 0.05 | 0.00-0.09 |
| Basophil (%) | 0.02↑ | 0.00-0.01 |
| Eosinophil, ×10^9^/L | 0.38 | 0.00-0.68 |
| Basophil, ×10^9^/L | 0.13↑ | 0.00-0.07 |
| Mean corpuscular volume (MCV), fl | 80.77 | 80-100 |
| Mean corpuscular hemoglobin (MCH), pg | 26.80 | 25-34 |
| Mean corpuscular hemoglobin concentration (MCHC), g/L | 331.80 | 310-355 |
| Hematocrit value (HCT) | 0.438 | 0.39-0.51 |
| Platelet hematocrit (PCT) | 0.11 | 0.11-0.27 |
| Total bile acid, umol/L | 9.40 | 0.00-10.00 |
| Total bilirubin (TBil), umol/L | 12.4 | 0-26 |
| Direct bilirubin (DBil), umol/L | 2.6 | 0-6.8 |
| Indirect bilirubin (IBil), umol/L | 9.8 | 3.1-14.3 |
| Aspartate aminotransferase (AST), U/L | 20 | 12-37 |
| Alanine aminotransferase (ALT), U/L | 10 | 7-43 |
| Alkaline phosphatase (ALP), U/L | 161 | 64-443 |
| Cholinesterase, U/L | 8386 | 5000-12000 |
| Creatine kinase (CK), U/L | 68 | 50-310 |
| CK-MB, U/L | 18 | 0-25 |
| Lactate dehydrogenase (LD), U/L | 191 | 120-250 |
| Lactic acid, mmol/L | 2.86↑ | 0.600-2.200 |
| Total protein, g/L | 65.90↓ | 68-88 |
| Albumin, g/L | 39.9↓ | 42-56 |
| Globulin, g/L | 26.0 | 19.0-40.0 |
| Prealbumin, mg/L | 313.30 | 200.00-430.00 |
| Urea nitrogen, mmol/L | 3.26 | 2.70-7.70 |
| Serum creatinine, umol/L | 60 | 52-101 |
| Uric acid, umol/L | 405 | 208-428 |
| Bicarbonate radical, mmol/L | 25.60 | 22.00-29.00 |
| Endogenous creatinine clearance rate, ml/min | 68.10↓ | 85-125 |
| Cystatin C, mg/L | 1.11↑ | 0.59-1.03 |
| Hypersensitive C-reactive protein, mg/L | <0.80 | 0-1 |
| C-reactive protein (CRP), mg/L | <10.00 | 0-10 |
| Immunoglobulin G (IgG), g/L | 9.80 | 8.6-17.4 |
| Immunoglobulin A (IgA), g/L | 0.01↓ | 1.0-4.2 |
| Immunoglobulin M (IgM), g/L | 3.27↑ | 0.3-2.2 |
| Immunoglobulin G4 (IgG4), g/L | 0.10 | 0.00-2.00 |
| Erythrocyte sedimentation rate (ESR), mm | 6 | 0-15 |
| Interleukin-6 (IL-6), pg/ml | 2.51 | 0-7 |
| Epstein-Barr virus PCR | negative | negative |
| Epstein-Barr virus VCA-IgG | negative | negative |
| Epstein-Barr virus nuclear antigen IgG | negative | negative |
| Herpes simplex  virus 1/2 PCR | negative | negative |
| Total cholesterol, mmol/L | 5.24 | <5.20 |
| Triglyceride, mmol/L | 1.04 | <1.70 |
| High-density lipoprotein, mmol/L | 1.36 | ≥1.00 |
| Low-density lipoprotein, mmol/L | 3.52 | <3.40 |
| Homocysteine (HCY), umol/L | 13.0 | 0-15 |
| Blood glucose, mmol/L | 4.38 | 3.9-6.1 |
| Blood kalium, mmol/L | 3.97 | 3.50-4.90 |
| Blood natrium, mmol/L | 140.3 | 135.00-145.00 |
| Blood chlorine, mmol/L | 104.70 | 98.00-110.00 |
| Blood calcium (Total), mmol/L | 2.53 | 2.10-2.80 |
| Blood magnesium, mmol/L | 0.85 | 0.50-0.90 |
| Blood phosphorus, mmol/L | 1.39 | 0.84-1.71 |

Results of laboratory tests 8 weeks after his second admission

| Laboratory indicators | Case (16yrs) | Normal range (age-matched) |
| --- | --- | --- |
| White blood cell (WBC), ×109/L | 7.14 | 4.1-11.0 |
| Red blood cell (RBC), ×109/L | 5.56 | 4.5-5.9 |
| Hemoglobin (HGB), g/L | 151.60 | 129.00-172.00 |
| Platelet (PLT), ×109/L | 231.20 | 150.00-407.00 |
| Neutrophil (%) | 0.54 | 0.37-0.77 |
| Lymphocyte (%) | 0.34 | 0.17-0.54 |
| Monocyte (%) | 0.08 | 0.02-0.11 |
| Neutrophil, ×109/L | 3.82 | 1.8-8.3 |
| Lymphocyte, ×109/L | 2.45 | 1.2-3.8 |
| Monocyte, ×109/L | 0.54 | 0.14-0.74 |
| Eosinophil (%) | 0.04 | 0.00-0.09 |
| Basophil (%) | 0.007 | 0.00-0.01 |
| Eosinophil, ×109/L | 0.28 | 0.00-0.68 |
| Basophil, ×109/L | 0.05 | 0.00-0.07 |
| Mean corpuscular volume (MCV), fl | 83.69 | 80-100 |
| Mean corpuscular hemoglobin (MCH), pg | 27.27 | 25-34 |
| Mean corpuscular hemoglobin concentration (MCHC), g/L | 325.80 | 310-355 |
| Hematocrit value (HCT) | 0.465 | 0.39-0.51 |
| Platelet hematocrit (PCT) | 0.175 | 0.11-0.27 |
| Aspartate aminotransferase (AST), U/L | 64↑ | 12-37 |
| Alanine aminotransferase (ALT), U/L | 79↑ | 7-43 |
| Creatine kinase (CK), U/L | 223 | 50-310 |
| CK-MB, U/L | 34 | 0-25 |
| Urea nitrogen, mmol/L | 3.98 | 2.70-7.70 |
| Serum creatinine, umol/L | 64 | 52-101 |
| Uric acid, umol/L | 406 | 208-428 |
| Lactic acid, mmol/L | 2.480↑ | 0.600-2.200 |
| Blood glucose, mmol/L | 5.10 | 3.9-6.1 |
| Blood kalium, mmol/L | 3.81 | 3.50-4.90 |
| Blood natrium, mmol/L | 138.5 | 135.00-145.00 |
| Blood chlorine, mmol/L | 107.4 | 98.00-110.00 |
| Blood calcium (Total), mmol/L | 2.33 | 2.10-2.80 |
| Blood magnesium, mmol/L | 0.83 | 0.50-0.90 |
| Blood phosphorus, mmol/L | 1.00 | 0.84-1.71 |
| Follicle stimulating hormone (FSH), mIU/ml | 5.60 | 1.50-12.40 |
| Luteinizing hormone (LH), mIU/ml | 4.61 | 1.40-7.70 |
| Prolactin, ng/ml | 27.08↑ | 3.00-16.50 |
| Progesterone, mg/ml | 0.15 | 0.10-0.66 |
| Testosterone, ng/ml | 3.32 | 1.37-8.57 |
| Estradiol, pg/ml | 31.10 | 0.00-84.00 |
| Epstein-Barr virus PCR | negative | negative |

Results of laboratory tests 8 weeks after his second admission

| Laboratory indicators | Case (16yrs) | Normal range (age-matched) |
| --- | --- | --- |
| White blood cell (WBC), ×109/L | 9.18 | 4.1-11.0 |
| Red blood cell (RBC), ×109/L | 5.57 | 4.5-5.9 |
| Hemoglobin (HGB), g/L | 155.40 | 129.00-172.00 |
| Platelet (PLT), ×109/L | 204.50 | 150.00-407.00 |
| Neutrophil (%) | 0.52 | 0.37-0.77 |
| Lymphocyte (%) | 0.37 | 0.17-0.54 |
| Monocyte (%) | 0.07 | 0.02-0.11 |
| Neutrophil, ×109/L | 3.81 | 1.8-8.3 |
| Lymphocyte, ×109/L | 2.59 | 1.2-3.8 |
| Monocyte, ×109/L | 0.43 | 0.14-0.74 |
| Eosinophil (%) | 0.03 | 0.00-0.09 |
| Basophil (%) | 0.005 | 0.00-0.01 |
| Eosinophil, ×109/L | 0.31 | 0.00-0.68 |
| Basophil, ×109/L | 0.04 | 0.00-0.07 |
| Mean corpuscular volume (MCV), fl | 84.01 | 80-100 |
| Mean corpuscular hemoglobin (MCH), pg | 27.90 | 25-34 |
| Mean corpuscular hemoglobin concentration (MCHC), g/L | 332.10 | 310-355 |
| Hematocrit value (HCT) | 0.468 | 0.39-0.51 |
| Mean platelet volume (MPV), fl | 7.37↓ | 9.00-12.00 |
| Platelet hematocrit (PCT) | 0.175 | 0.11-0.27 |
| Blood glucose, mmol/L | 5.10 | 3.9-6.1 |
| Blood kalium, mmol/L | 4.36 | 3.50-4.90 |
| Blood natrium, mmol/L | 140.3 | 135.00-145.00 |
| Blood chlorine, mmol/L | 104.8 | 98.00-110.00 |
| Blood calcium (Total), mmol/L | 2.31 | 2.10-2.80 |
| Blood magnesium, mmol/L | 0.87 | 0.50-0.90 |
| Blood phosphorus, mmol/L | 1.26 | 0.84-1.71 |
| Antistreptolysin O (ASO), IU/ml | 20 | ≤170 |
| Rheumatoid factor (RF), IU/ml | 19.80 | ≤18 |
| Epstein-Barr virus PCR | negative | negative |
| Immunoglobulin G (IgG), g/L | 10.26 | 8.6-17.4 |
| Immunoglobulin A (IgA), g/L | 0.01↓ | 1.0-4.2 |
| Immunoglobulin M (IgM), g/L | 2.62↑ | 0.3-2.2 |
